# Scalable Database Indexing and Fast Image Retrieval Based on Deep Learning and Hierarchically Nested Structure Applied to Remote Sensing and Plant Biology

**DOI:** 10.3390/jimaging5030033

**Published:** 2019-03-01

**Authors:** Pouria Sadeghi-Tehran, Plamen Angelov, Nicolas Virlet, Malcolm J. Hawkesford

**Affiliations:** 1 Department of Plant Sciences, Rothamsted Research, Harpenden AL5 2JQ, UK; 2 School of Computing and Communications, InfoLab21, Lancaster University, Lancaster LA1 4WA, UK

**Keywords:** content-based image retrieval, deep convolutional neural networks, information retrieval, data indexing, recursive similarity measurement, deep learning, bag of visual words, remote sensing

## Abstract

Digitalisation has opened a wealth of new data opportunities by revolutionizing how images are captured. Although the cost of data generation is no longer a major concern, the data management and processing have become a bottleneck. Any successful visual trait system requires automated data structuring and a data retrieval model to manage, search, and retrieve unstructured and complex image data. This paper investigates a highly scalable and computationally efficient image retrieval system for real-time content-based searching through large-scale image repositories in the domain of remote sensing and plant biology. Images are processed independently without considering any relevant context between sub-sets of images. We utilize a deep Convolutional Neural Network (CNN) model as a feature extractor to derive deep feature representations from the imaging data. In addition, we propose an effective scheme to optimize data structure that can facilitate faster querying at search time based on the hierarchically nested structure and recursive similarity measurements. A thorough series of tests were carried out for plant identification and high-resolution remote sensing data to evaluate the accuracy and the computational efficiency of the proposed approach against other content-based image retrieval (CBIR) techniques, such as the bag of visual words (BOVW) and multiple feature fusion techniques. The results demonstrate that the proposed scheme is effective and considerably faster than conventional indexing structures.

## 1. Introduction

Today, digital images and videos are ubiquitous in every domain. The advancement in multi-media technologies has led to the generation of an enormous number of images and videos. The size of image repositories has increased rapidly in many domains, such as biology, remote sensing, medical, military, and web-searching. The use of automated data acquisition systems, such as modern phenotyping platforms [1,2,3] has revolutionized the way the data is collected and analyzed. The plant science community is seeking novel solutions to fully exploit all the potential offered by such new platforms equipped with high-resolution remote sensing sensors. Any large-scale dataset in modern biological sciences first and foremost requires reliable data infrastructure and an efficient information retrieval system. For image repositories of large scale, manual tagging is infeasible and is prone to errors, due to users’ subjective opinions. Thus, to utilize such unstructured and complex image collections, there is a substantial need for content-based image retrieval (CBIR) systems for browsing through images at a large scale and to classify, structure, and retrieve relevant information requested by the users. 

Information retrieval (IR) refers to finding material (image repositories or documents) of an unstructured nature (image or text) that satisfies an information need from within large collections [4]. There is a fundamental difference between CBIR and search by text and metadata. Searching methods based on metadata rarely examine the content of an image itself but rather rely on manual annotations and tagging. In these systems, words are stored as ASCII character strings to describe image content. However, the high complexity of images cannot be described easily by keywords; thus, retrieval systems which are based solely on manual annotation often lead to unsatisfactory outcomes. In contrast, CBIR does not require keywords (manual annotation) and desired images are retrieved automatically based on their similarity to the query representation [5,6,7].

Although CBIR techniques are beginning to find a foothold in many applications, such as biology, remote sensing, satellite imaging, etc., the technology still suffers from lack of maturity due to a significant gap towards semantic-aware retrieval from visual content. A major challenge associated with CBIR systems is to extract information from an image which is unique and representative, to overcome the issue of so called *semantic-gap*. The *semantic-gap* refers to low-level features of images such as colors and texture, but those features might not be able to extract a higher level of understanding of the image perceived by humans [8]. Due to the absence of solid evidence on the effectiveness of CBIR techniques for high-throughput datasets with varied collections of images, opinion is still sharply divided regarding the reliability and performance of such systems in real-time. It is essential to standardize CBIR for easy access to data and speed up the retrieval process.

In this paper, a new concept of CBIR is employed to exploit the opportunities presented by large image-based repositories, particularly in remote sensing and plant biology. The proposed approach, which relies solely on the contents of the images, will pave the way for a computationally efficient and real-time image querying through an unstructured image database. An end-to-end CBIR framework is conducted without supervision. First, we utilize a deep CNN model as a feature extractor to obtain the feature representations from the activations of the convolutional layers. In the next step, a hierarchically nested database indexing structure and local recursive density estimation are developed to facilitate an efficient and fast retrieval process. Finally, the key elements of CBIR, accuracy and computational efficiency, are evaluated and compared with the state-of-the-art CBIR techniques.

## 2. Related Works

The core modules of any CBIR systems include image representation, database indexing, and image scoring, described as detailed below: 

### 2.1. Image Representation

At the core, visual features affect every aspect of computer vision applications, including CBIR. The success of any CBIR system crucially depends on the feature representation of the images extracted by applying an image descriptor. Although over the past decades a variety of feature extraction techniques have been developed to find semantically richer image representations, it still remains one of the key challenges in CBIR applications.

#### 2.1.1. Hand-crafted Feature Extraction Techniques

Handcrafted features are used excessively in conventional CBIR applications to quantify the contents of images. Earlier applications mainly focused on primitive features (global features) which describe an image as a whole to generalise the entire image as a single vector, such as contour representations, texture, or shape features.

- Color properties are extracted directly from the pixel densities over the whole image, segmented regions/bins, or sub-image. Image descriptors that characterize the color properties of an image seek to model the distribution of the pixel intensities in each channel of the image. These methods include color statistics, such as deviation, mean, and skewness, along with color histograms. Since color features are robust to background complications and are invariant to the size or orientation of an image, the color based methods have become one of the most common techniques in CBIR [9,10,11].
- Texture properties measure visual patterns in images that contain important information about the structural arrangement of surface i.e., fabric, bricks, etc. Texture descriptors seek to model the feel, appearance, and overall tactile quality of an object in an image and are defined as a structure of surfaces formed by repeating a particular element or several elements in different relative spatial distribution and synthetic structure. In general, the repetition involves local variations of scale, orientation, or other geometric and optical features of the elements [12,13].
- Shape properties can also be considered as one of the fundamental perceptual characteristics. Shape properties take on many non-geometric and geometric forms, such as moment invariants, aspect ratio, circularity, and boundary segments. There are difficulties associated with shape representation and descriptors techniques due to noise, occlusion, and arbitrary distortion, which often causes inaccuracies in extracting shape features. Nonetheless, the method has shown promising results to describe the image content [14,15].

Whilst the above techniques focus on primitive features, more recent techniques have been aimed to find semantically richer image representations by extracting a collection of local invariant features. The main advantage of semantic features is locality, which means that the extracted features are local and robust to clutter and occlusion. Also, individual features can be matched to a large database of objects and have close to real-time performance.

One of the most effective techniques is the bag of visual words technique [16,17]. The main reasons that BOVW has gained popularity in classification and retrieval applications are the use of powerful local descriptors, such as Scale Invariant Feature Transform (SIFT) [18], Speeded Up Robust Features (SURF) [19], and Binary Robust Invariant Scalable Keypoints (BRISK) [20]. In addition, the vector representations can be compared with standard distances, and subsequently be used for effective CBIR. However, the main drawback of BOVW is the high dimensional vector representing an image. Although a high-dimensional vector usually provides better exhaustive search results compared to a low-dimensional one, it is more difficult to index efficiently. Aggregated vectors, such as Fisher Vector (FV) [21] and Vector of Locally Aggregated Descriptors (VLAD) [22] aim to address this problem by encoding an image into a single vector, reducing the dimensionality without noticeably impacting the accuracy [16,17].

Nevertheless, despite the robustness of local descriptors techniques, global features are still desirable in a variety of computer vision applications. Ultimately, having an intimate knowledge of the dataset contents will provide a better perspective for which feature extraction techniques might be appropriate. For example, datasets that have relatively different color distributions, color descriptors will be more effective. Nonetheless, the effectiveness of hand-crafted feature representation in CBIR is inherently limited, as these approaches mainly operate at the primitive level. As presented in the following section, higher accuracy will be achieved by extracting semantic features from images based on learning-based features using deep networks.

#### 2.1.2. Learning-based Features Using Deep Convolutional Neural Network

Recent years have witnessed the success of learning based features using Deep Neural Networks (DNNs) [23,24,25]. Unlike conventional global and local feature extraction methods, which often use shallow architecture and solely rely on human-crafted features, deep Conventional Neural Networks are considered the most well-known architecture for visual analysis [26]. CNN models attempt to model high-level abstractions in images by employing deep architectures composed of multiple non-linear transformations [27]. In CNNs, features are extracted at multiple levels of abstracts and allow the system to learn complex functions that directly map raw sensory input data to the output, without relying on hand-engineered features using domain knowledge. 

CNN has achieved state-of-the-art performance in a variety of applications, including natural language processing [28,29], speech recognition [30], and object recognition [31]. Inspired by the success of CNN in many computer vision applications, it has started to gain a foothold in the research area of CBIR. Subsequently, CNN models have been proposed to improve the image retrieval workflow [16,32,33]. For instance, in Sun et al. [34], features derived from local image regions identified with a general object detector and an adapted CNN model have been evaluated on two public large-scale image datasets. Lai et. al. [35] proposed simultaneous feature learning using deep neural networks and hash coding. The short binary codes resulted from hash coding achieved efficient retrieval and a considerable saving in memory usage. In other techniques, CNN descriptors are combined with conventional descriptors such as the VLAD representation [36,37]. Finally, in Mohedano et al. [38] authors proposed a method based on encoding the convolutional features of CNN and the BOVW aggregation scheme. The approach outperformed the state-of-the-art tested on landmark datasets.

### 2.2. Feature Indexing and Image Scoring

Another line of research in CBIR focuses on feature indexing and structuring the data vectors extracted from images. Feature indexing refers to structuring a database to facilitate search speed. Since one of the key features in CBIR systems is response time, the importance of feature indexing becomes more vivid, especially in a large-scale image database. An efficient database indexing can significantly accelerate the retrieval process and reduces memory usage substantially [39]. Conventional methods use a similarity metric to compare the feature vector of the query image to each and every single feature vector in the database. However, whilst comparing the query feature vector to the entire image dataset might be feasible for small datasets, this is still an *O(N)* linear operation; thus, for large-scale datasets of billions of feature vectors, this is not computationally efficient. In [39,40], a hierarchical structure is formed based on low-level feature extraction techniques such as color, texture, and local mean clustering technique. The model is problem-specific and threshold-dependent. The main drawbacks are that the developed primitive features are not effective enough to represent images; in addition, the wrong choice of cluster radius may have a negative impact on the retrieval performance. 

Two widely used indexing techniques in CBIR are inverted file index and hashing based indexing. An inverted index (also called “inverted file”) is the central component of many search systems [41,42,43,44,45] as it facilitates faster and more scalable querying. Inspired by the field of information retrieval (i.e., text search engine), the inverted index stores mapping of unique word IDs to the document IDs in which the words occur [45]. It is easy to conceptualize an inverted index as a dictionary data structure with the word ID as the key and the value as a list of document IDs that contain the word.

The hashing based index projects images into a common Hamming space, while similar data will be mapped into similar binary codes [46,47,48,49]. The main concern of the existing hashing scheme such as locality sensitive hashing (LSH) [50] is an expensive memory cost. The reason is that these methods require to store the raw dataset representation vectors in memory, which is not scalable for a large-scale image database. Inspired by the success of deep networks, deep hashing methods have been proposed for image retrieval systems to take advantage of the deep network’s image representation power [35,46,47,51].

## 3. Methodology

**Figure 1:**
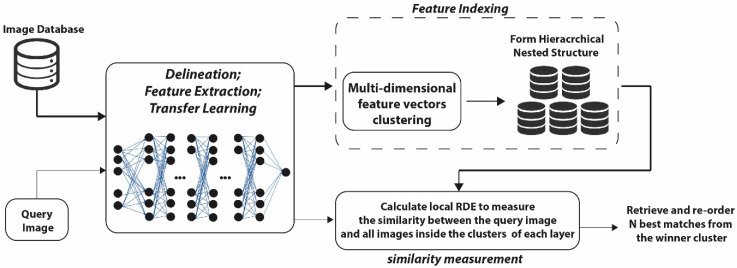
Schematic representation of the retrieval model.

In this paper, we focus on three key challenges of any content-based image retrieval: image representation, database indexing, and image similarity measurement. Figure 1 illustrates an overall view of the proposed framework. The first step in the prescriptive analytics process is to transform the initial unstructured and structured data sources into analytically prepared data. To achieve a balance between complexity and efficiency, a pre-trained CNN is used to utilize the ability of the model to produce better image representations for the retrieval task. We leverage an existing model trained on the ImageNet dataset [52], known as residual network (ResNet) [53]. The model is used as a fixed feature extractor without the last fully connected layer. The trained model provides access to the visual descriptors previously learnt by the CNN after processing millions of images in the ImageNet dataset without requiring a computational expensive training phase. 

Although the deep learning model is effective in extracting discriminative visual features from images (Section 4.2), it would compute multi-dimensional feature vectors (2048-D in our case) for every image which increases the computational complexity for feature indexing and querying. To address the multi-dimensional complexity caused by the CNN model, a novel nested hierarchical database indexing is proposed to facilitate fast querying. In addition, a recursive calculation based on local density estimation is used to measure the similarity between the given query and all the images from a given image cluster.

### 3.1. Representation Learning Using Residual Learning Model

**Figure 2:**
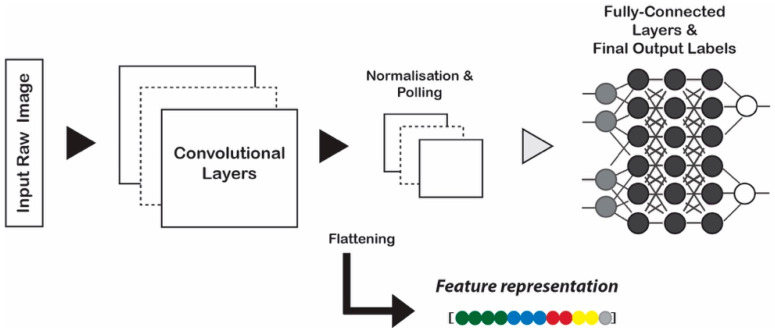
Representation learning scheme. Deep feature extraction from the pretrained Convolutional Neural Network (CNN) model.

CNNs process images through several layers, mainly in two parts of (a) the convolutional layers and max pooling layers and (b) the fully connected layers which are typically a linear classifier, such as softmax regression classifier (Figure 2). The convolutional layers are used to detect features whereas normalization and pooling layers control overfitting and reduce the number of weights. The last fully-connected layers are used for classification. Recent studies [23,54] indicate that it is feasible to adapt CNN models to extract semantic aware features by the activation of different layers in the networks [52]. Such generic descriptors derived from CNN are effective and powerful.

As mentioned, Neural Networks have the ability to learn and discover a good combination of features, even for complex tasks which would otherwise require a lot of human effort to be manually hand-crafted. In practice, it is common to pre-train a CNN on a very large dataset such as ImageNet dataset with 1.2 million images and 1000 categories, and then use the model either as an initialization for fine-tuning the CNN or use it as a fixed feature extractor, which is also known as *Representation Learning (RL)*. The main reason is that it is relatively rare to have a dataset big enough to train an entire CNN from scratch; additionally, training a CNN model from scratch will take considerable time to train across multiple GPUs on a large-scale dataset such as ImageNet. 

Representation learning is the improvement of learning in a new task through the transfer of knowledge from a related task that has already been learned [55]. In such a model, an existing pre-trained model is used as a starting point for a new task, such as classification. The conventional CNNs are treated as end-to-end image classifiers where an image forward propagates through the network and the final probabilities are obtained from the end of the network. However, in the *representation learning*, instead of allowing the image to forward propagate through the entire network, we can stop the propagation at an arbitrary layer, such as the last fully connected layer, and extract the values from the network at this time, and then use them as feature vectors. 

In this study, we utilize the convolutional layers merely as a feature extractor. The aim is to generalize a trained CNN in learning discriminative feature representations for the images in our dataset. The trained model is used to derive feature vectors, more powerful than hand-designed algorithms such as SIFT, GIST, HOG, etc. We exploit the ability of a well-known deep convolutional neural network framework known as residual learning (ResNet) [53,56]. Residual learning frameworks ease the training of deeper networks and are a great candidate to capture the discriminative properties of images as a fixed feature extractor model. Network depth is a key element in neural network architecture; however, deeper networks are more difficult to train, as the accuracy gets saturated and then degrades rapidly. When deeper networks start converging, a degradation problem is exposed which is not caused by overfitting, while adding more layers causes even higher training error. In residual learning models, instead of learning a direct mapping of x→y with a function H(x), the residual function is defined using H(x)=F(x)+x; where F(x) and *x* represents residual mapping function and the identity function, respectively. The author’s hypothesis is that it is easier to optimize F(x) than to optimise the original mapping function, H(x). We refer readers to [53,56] for more details.

The employed ResNet model has been pre-trained on the ImageNet Large Scale Visual Recognition Challenge (ILSVRC) 2012, to classify 1.3 million images to 1000 ImageNet classes [52]. The ResNet consists of convolutional layers, pooling layers, and fully connected layers. The network takes images of size 224 × 224 pixels as input then passes through the network in a forward pass after applying filters to the input image. When treating networks as a fixed feature extractor, we cut off the network at an arbitrary point (normally prior to the last fully-connected layers); thus, all images will be extracted from the activations of convolutional feature maps directly. This would compute a 2048-D feature vector for every image that contains the hidden layer immediately before the classifier. The 2048-D feature vectors will be directly used for computing the similarity between images. The computational complexity and retrieval process may become cumbersome as the dimensionality grows. This requires us to optimize the retrieval process by proposing a hierarchically nested indexing structure and recursive similarity measurements to facilitate faster access and comparison of multi-dimensional feature vectors as described in the following sections.

### 3.2. Feature Indexing Based on Hierarchical Nested Data Clusters

The success of a CBIR not only depends on image delineation, but feature indexing and similarity measurement matrix also play vital roles to facilitate the execution of queries. In general, feature indexing refers to a database organizing structure to assist fast retrieval process. Whilst it is feasible to retrieve information from datasets which are small in size by measuring the similarity between a query and every image in the dataset, the computational complexity will soon increase significantly on a larger scale image database. 

**Figure 3:**
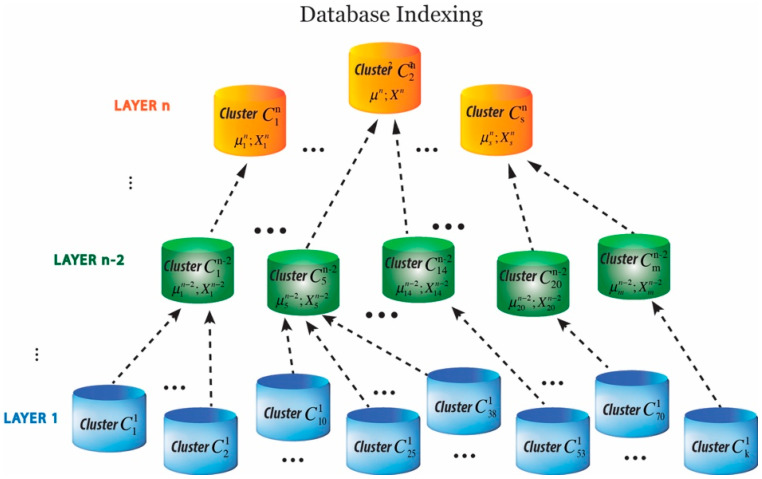
Schematic representation of the hierarchical nested indexing structure.

In an attempt to address the challenges faced by retrieval information on a large-scale dataset, we present a hierarchically nested structure. The introduced database indexing aims at arranging and structuring the image database into a simple yet effective form of data clusters and hierarchies. Although forming a hierarchical structure for retrieval optimization has been explored before [57,58,59,60], the method presented in this study is quite different. Hierarchically nested data clusters are structured in which data clusters at higher layers represent one or multiple clusters at a lower layer based on mean values of the cluster centers (Figure 3). The first layer clusters are generated based on feature representations derived from the CNN model. Data clusters are formed by grouping the relevant data points using a partition-based clustering approach known as *K*-means clustering [61]. Figure 3 illustrates how the hierarchical structure of clusters is formed. µ and *X* are abstract values and denote mean values and scalar products explained in Section 3.3. 

### 3.3. Fast Searching and Similarity Measure Based on Recursive Data Density Estimation

**Figure 4:**
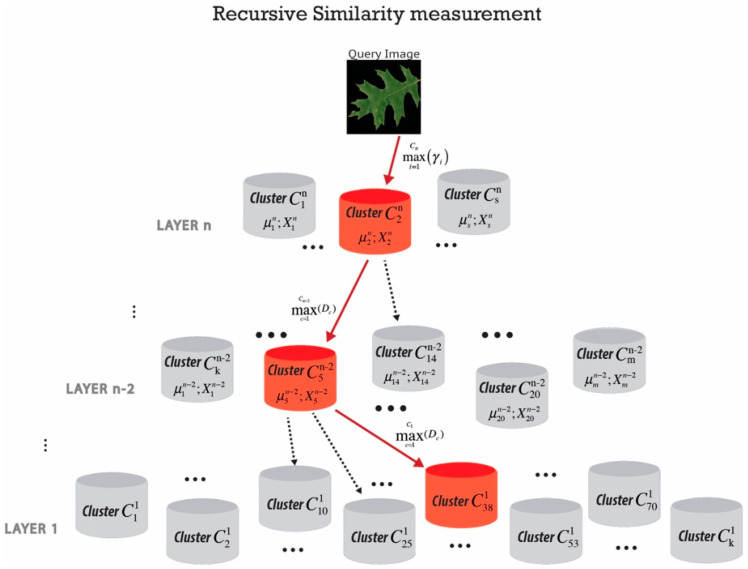
Schematic representation of searching through hierarchical nested structure and retrieve the most similar images (winner cluster) to the query.

The final step after forming the hierarchically nested data clusters is to find the cluster which contains the most similar images to a query image. We applied recursive density estimation [62,63] to measure a similarity between the query image and all images inside each cluster recursively. The main idea of the recursive density function is to estimate the probability density function by a Cauchy type kernel and to recursively calculate it. The method is also applied for novelty detection in real-time data streams and video analytics [64]. The recursive calculation allows us to discard each data once it has been processed and only store the accumulated information in memory concerning the local mean (per cluster), µ and scalar product X. In order to speed up the retrieval process by an order of magnitude, the searching process is performed from the top of the pyramid in an ordered hierarchy based on “winner takes all” principle with maximum local recursive density estimation at each level (Figure 4). 

The degree of similarity between the query image to images inside each cluster is measured by the relative local density with regards to the query image, which is defined by a suitable kernel over the distance between the current image sample and all the other images inside the cluster:

(1) $$D_{i}^{c}=K\left(\overset{M^{c}}{\underset{j=1}{\sum }}d_{ij}^{c}\right)\;c=\left[1,C\right]$$

where $M^{c}$ is the number of images associated with *c^th^* cluster; $d_{ij}^{c}$ denotes the distance between the query image and any other image of the *c^th^* cluster; $i=1,2,\ldots ,N_{c}$; *N* is the number of images within *c^th^* cluster.

Different types of distance measures can be used, such as Euclidean or Cosine distance. We used a Cauchy type of kernel to define the local density $D_{i}^{c}$. It can be proven that Cauchy type kernel asymptotically tends to Gaussian, but can be calculated recursively [63]:

(2) $$D_{i}^{c}=\frac{1}{1+∥F_{i}-\mu_{i}^{c}{∥}^{2}+X_{i}^{c}-∥\mu_{i}^{c}{∥}^{2}}$$

$F=\left\{f_{1},\cdots ,f_{2048}\right\}$ is the feature vector. $i=1,2,\ldots ,N_{c}$; *N_c_* is the number of images within *c^th^* cluster.

Both the mean, *µ_i_* and the scalar product, *X_i_* are updated recursively as follows [63]:

(3) $$\mu_{i}=\frac{i-1}{i}\mu_{i-1}+\frac{1}{i}F_{i};\mu_{1}=F_{1}$$

(4) $$X_{i}=\frac{i-1}{i}X_{i-1}+\frac{1}{i}∥F_{i}{∥}^{2};X_{1}=∥F_{1}{∥}^{2}$$

Finally, the cluster with the maximum local density *D^c^*, with respect to the query image, is most likely to contain similar images:

(5) $$C_{i}^{*}=argmax_{c=1}^{C}\left\{D_{i}^{c}\right\}$$

The final step is the similarity measurement between the query image and all the images inside the winning cluster at the lowest layer. The relevance score is defined by distance-based scoring using City Block distance. Images are then ranked accordingly to their obtained scores. A smaller value of City Block distance implies that the corresponding image is more similar to the query image and vice versa. The City Block distance between the query image and images inside the winner cluster is calculated as follows:

(6) $$d\left(I^{j},Q\right)=\overset{K}{\underset{k=1}{\sum }}\left|Q_{k}-I_{k}^{j}\right|;j=1,\ldots ,N_{c}$$

where *N_c_* is the number of images of winning cloud; *K* is the number of extracted features (*K* = 2048); *Q* denotes the query image; and *I* is the image in the winning cluster.

## 4. Experiments and Results

In this section, we present the experiments conducted to evaluate the key elements of CBIR: accuracy and computational efficiency. The deep learning framework and feature indexing were developed in Python using Keras API and MATLAB, respectively. The experiment was carried out on a desktop PC with Intel Core i7, processing power with 3.4 GHz CPU, 24 GB RAM, and GeForce GT 640 GPU running Ubuntu 16.04. Furthermore, the accuracy of the proposed approach is compared with two hand-crafted feature-based methods, known as BOVW and multiple fused global features (MFF). In addition, the computational efficiency and retrieval execution timing are evaluated against inverted file indexing and non-hierarchical searching.

Integrating multiple features: As mentioned, correct selection and utilizing appropriate features to represent an image are key elements for having a more accurate retrieval system [65,66]. The common approach is to combine color and texture properties to generate a robust feature representation [66,67]. In this study, two feature extractor techniques based on color and texture properties known and color correlogram and GIST are integrated. GIST descriptor [68] is widely used in scene classification to represent an image by a vector of spectral values which is based on spatial envelope properties, such as ruggedness, expansion, naturalness, and roughness. Color auto-correlogram, on the other hand, is used to preserve the spatial information of colors in an image. It describes the global distribution of local spatial correlations between identical colors [69].

Bag of visual words: In the BOVW method, the SIFT algorithm [18] is applied as a feature descriptor in addition to Local Linear Constraint (LLC) [70] to project the descriptors into the visual vocabulary and to reduce the computational complexity. In addition, to preserve the spatial relationships of the code vector, Spatial Pyramid Matching (SPM) [71] was developed where the entire image was divided into levels. Each image is divided into spatial sub-regions and histograms of features are computed from each sub-region. Each level divides the image into $2^{l}\times 2^{l-1}$; where *l* is level. The features are computed locally for each grid and the spatial information is incorporated into histograms. A three-level SPM is comprised of a single histogram in level 0, 4 histograms in level 1, and 16 histograms in level 2. In the end, the histograms from all the sub-regions are concatenated together to generate the final representation of the image. The result is a feature vector of (1 + 4 + 16) × *K*; where *K* is the number of codebooks (*K* = 2000).

### 4.1. Datasets

**Figure 5:**
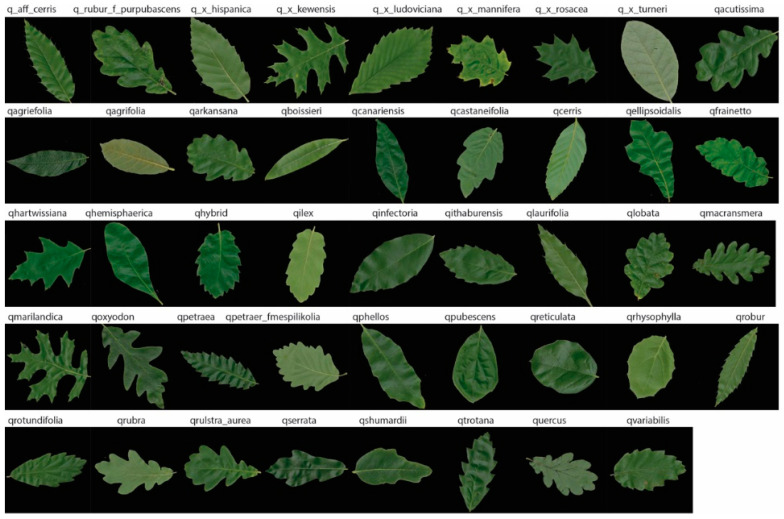
Sample images of MalayaKew 44 leaf collection.

*MalayaKew (MK) Leaf-Dataset*: This dataset [72] consists of a collection of leaves from 44 species class, with 52 images in each class. The data is in the form of digital images, size 256 × 256 pixels, collected at the Royal Botanic Garden, Kew, England. The dataset has been used solely for supervised image classification, since the dataset is extremely challenging as some of the classes have very similar appearances (Figure 5) making it extremely difficult to distinguish differences between classes with a fully unsupervised model, as was presented in this study. Although the MK dataset is not considered a big dataset, we believe the similarity between classes can be a good example to demonstrate how discriminative the features are between the convolutional neural networks and the hand-crafted methods.

**Figure 6:**
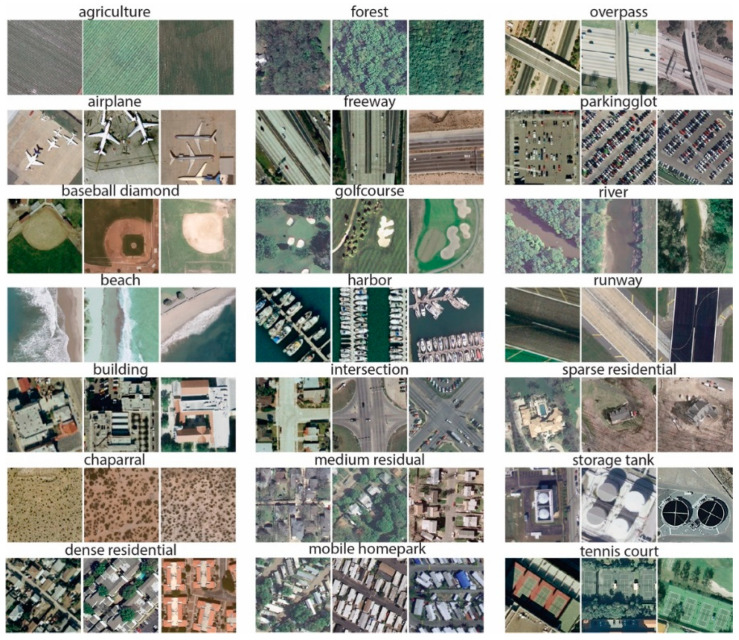
Sample images of the University of California Merced (UCM) dataset.

*The University of California Merced (UCM) Dataset*: UCM dataset [73] consists of 21 land cover, large-scale aerial images from the USGS national map urban area imagery. Each class contains 100 images with 256 × 256 pixels; the spatial resolution of each pixel is 30 cm measured in the RGB spectral space. The dataset has been widely utilized for evaluating the performance of high-resolution remote sensing image scene classification [74,75,76]. The UCM dataset shows very small inter-class diversity among some categories that share a few similar texture patterns or objects, which makes this dataset very challenging. Some sample image scenes from the UCM dataset are shown in Figure 6.

### 4.2. Performance and Accuracy

Throughout this work, we use two evaluation metrics widely used to assess CBIR performance, known as mean Average Precision (*mAP*) and the precision at rank *N* (*P*@*N*). Average Precision (*AP*) is one of the most frequent methods used to evaluate the retrieval quality of a single query’s retrieval results. *AP* takes consideration of both Precision (*Pr*) and Recall (*Re*). Precision is the fraction of retrieved images that are relevant, whereas Recall is the fraction of relevant images that are retrieved. *AP* averages the precision values from the rank positions where relevant images are retrieved. The mean average precision (*mAP*) is widely used to summaries the retrieval quality, which averages the *AP* over all queries. The definition of the above metrics follows below [4]:

(7) $$AP=\frac{\sum_{k=1}^{n}P\left(k\right)\times rel\left(k\right)}{R}$$

where *P(k)* denotes the precision of top *k* retrieval results; *rel(k)* is a binary indicator function equaling 1 if the *k^th^* retrieved results are relevant to the current query image and 0 otherwise; and *R* and *n* denote the number of relevant results for the current query image and the total number of retrieved results, respectively. Also, the precision at particular rank-*N* accuracy is another evaluation metric to evaluate CBIR performance. *P@N* score refers to the average number of same retrieved images, within the top-*N* ranked images. It should be noted that although *mAP* and *P@N* are widely used as evaluation metrics in CBIR, defining a suitable metric to measure the quality of results for an arbitrary query image is not a trivial process. In CBIR, it is hard to define the ground-truth since different users might have a different measure of similarity. If the degree of similarity of some of the images is very low, ignoring or not displaying those images is not critical and does not impact the overall performance of the system. Labelling images as non-relevant is not always satisfactory to the users. Any CBIR should have a certain tolerance for false positives, which often provides useful information.

In this study, to form a hierarchically nested pyramid, at the lower layer, images were grouped into a fixed number of clusters, while at the second layer, the means of the clusters at the first layer were further grouped into smaller numbers of clusters. Since the number of images in both datasets is in the region of few thousands, two-layer hierarchies are enough to achieve real-time image querying. In MK and UCM datasets, based on our experience, the number of clusters at the first layer was set to 44 and 21 clusters (number of categories) and 10 and 4 clusters at the top layer, respectively.

The retrieval process begins by calculating the local recursive density estimation between the query image and all the clusters at the top layer and selecting the winning cluster with maximum local Recursive Density Estimation (RDE). The search continues at the lower layers, but only with the clusters which associated to the winning cluster at the top layer. Finally, images in the winning cluster at the lowest stage are ranked based on calculating the eigenvector distance to the query image. 

#### 4.2.1. Retrieval Performance on MalayaKew Leaf-Dataset

**Figure 7:**
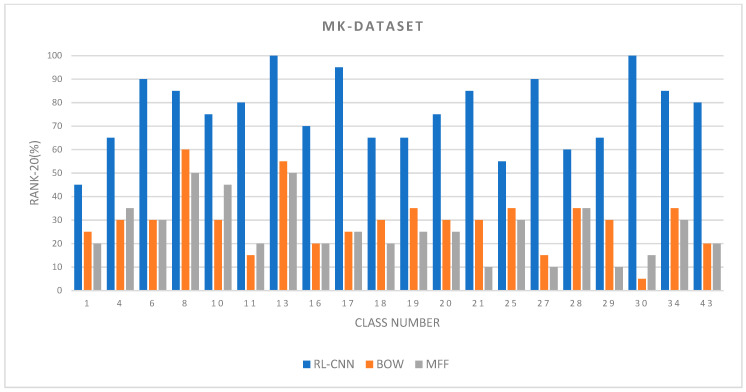
The retrieval Rank-20 accuracy between the Convolutional Neural Network (CNN) as a feature extractor, bag of visual words, and multiple feature fusion (color and texture).

The results of the convolutional neural network as a feature extractor (RL-CNN) are shown in Figure 7 and Table 1. The precision accuracy at rank-20 is compared in Figure 7 based on 20 queries. The queries were selected to tackle every range of visual appearances with a unique shape, such as *qoxyodon*, or similar appearances, like *q-aff-cerris* and *qlaurifolia.*

**Figure 8:**
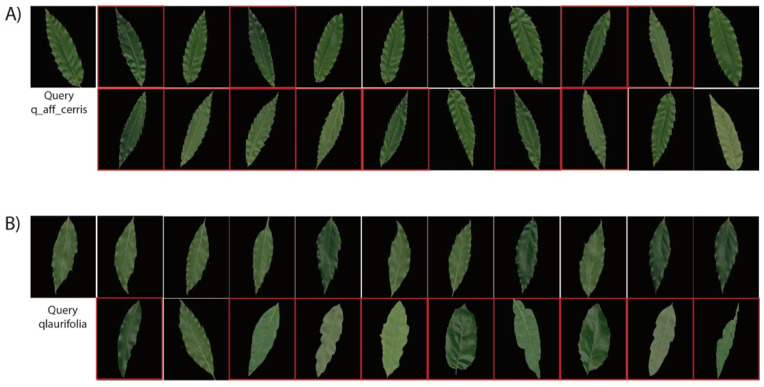
Qualitative evaluation of the proposed image retrieval on the two lowest performance of classes in Malaya–Kew Leaf-Dataset (**A**) Retrieval result from qlaurifolia class (**B**) retrieval result from q-aff-cerris class. The first image is the query and the following images are the images most similar to the query image. The retrieved images wrongly categorized are highlighted in red.

Several observations can be achieved from the precision results. The RL-CNN method outperformed the two state-of-the-art techniques by a large margin. The proposed method not only performed well on classes with unique visual appearances, such as *qlobata* or *qpetraea*, but it also distinguished categories with similar appearances, such as *quercus* and *q-x-kewensis*. In RL-CNN method, *q-x-mannifera*, *qboissieri*, *qellipsoidalis*, *qmacransmera*, and *qpetraea* obtained maximum accuracy with over 90%, whereas *qlaurifolia* and *q-aff-cerris* had the lowest value of 55% and 45%, respectively. The *qlaurifolia* class achieved 55% accuracy, whereas 9 out of 20 images belong to *qcanariensis*, *qrhysophylla*, and *qtrotana* categories (Figure 8A). The accuracy dropped to 35% and 30% in BOVW and MFF, accordingly. The *q-aff-cerris* class obtained the lowest accuracy in RL-CNN with 45% accuracy rate, whereas 11 out of 20 images belong to *qrobur* category, which is visually almost identical to the query image.

On the other hand, the BOVW and MFF performed poorly in identifying small differences between leaf varieties in MK dataset. Both methods retrieved images with the visual similarity to queries; however, they failed to distinguish small visual differences among classes. As illustrated in Figure 7, BOVW performed better than MFF in most cases, except classes *q_rubur_f_purpubascens, qagriefolia, qagrifolia*, and *qpetraea*. (The results for each class are presented in the Supplementary Materials).

Table 1 summarizes the *mAP* evaluation of the Malaya–Kew leaf dataset. The results are obtained from 20 queries in which the retrieval system can be tested and evaluated. The best accuracy score is 88.1%, achieved by RL-CNN, followed by BOVW and MFF with 66.2% and 52.6%, respectively.

#### 4.2.2. Retrieval Performance on UCM Dataset

**Figure 9:**
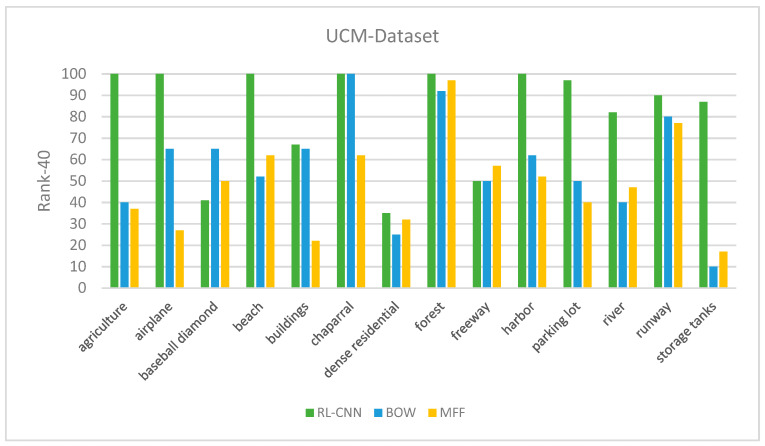
The retrieval Rank-40 accuracy between feature extractor using convolutional neural network, bag of visual words, and multiple feature fusion (color and texture).

**Figure 10:**
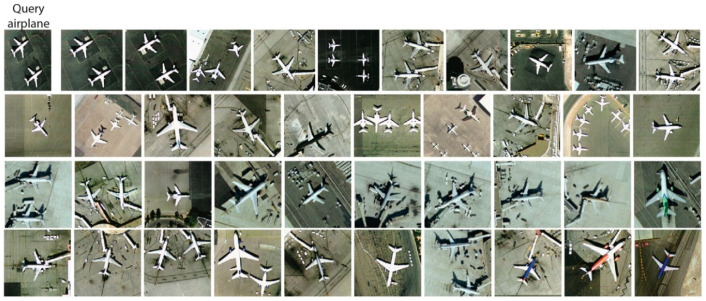
Retrieval results of airplane category using convolutional neural network as a feature extractor (RL-CNN). The methods obtained 100% retrieval accuracy.

**Figure 11:**
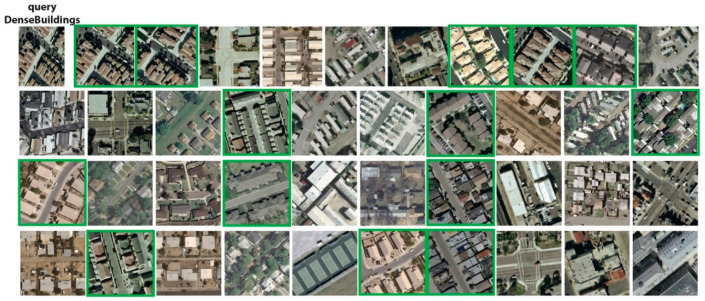
Retrieval results of dense-building category using convolutional neural network as a feature extractor (RL-CNN). The green rectangles indicate correct retrieval results.

**Figure 12:**
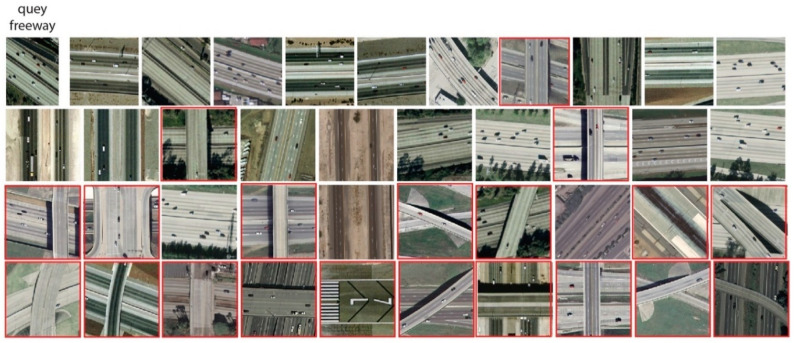
Retrieval results of freeway category using convolutional neural network as a feature extractor (RL-CNN). The red rectangles indicate incorrect retrieval results.

The precision performances at *P*@40 for the UCM dataset are shown in Figure 9. The results show that RL-CNN method outperformed both the BOW and MFF by achieving better accuracy in all categories except *baseball diamond* category. In RL-CNN, high accuracy results obtained in *agricultural*, *beach*, *forest*, *harbor*, *chaparral*, and *airplane* (Figure 10) categories. On the other hand, in the *baseball-diamond*, *dense residential* (Figure 11), and *freeway* (Figure 12), RL-CNN achieved the lowest accuracy with 41%, 35%, and 50%, respectively (Figure 9).

Figure 11 shows the retrieval results of the *dense building* class on a randomly given query. The class achieved 35% accuracy, whereas 14 out of 40 images belong to the same class as the query image. However, the rest of the images are still visually similar to the query retrieved from *medium residential* and *mobile home park* classes. The *freeway* class with 50% accuracy has a similar performance, whereas half of the retrieved images belong to *runway* and *overpass* classes, which are still visually very similar to the *freeway* class (Figure 12).

The retrieval *mAP* of different models on the UCM image dataset are listed in Table 1. As shown in the table, the RL-CNN outperformed both the state-of-the-art techniques. The *mAP* measure in RL-CNN is 90.1%, whereas the BOW and MFF achieved 86.2% and 69.8%, respectively.

### 4.3. Retrieval Time Per Query

Commercial CBIR applications are often assessed for requirements in computational capacity and memory efficiency. As mentioned earlier, the proposed hierarchically nested structure is beneficial for the retrieval performance in terms of search time and memory size required to store the indexed images. In this section, we provide more details on the retrieval time per query between the proposed method, the inverted index file method, and the non-hierarchical searching with a single layer. The non-hierarchical searching technique processes each image by scanning all image patches and computing similarity values for every individual image, unlike the nested hierarchical indexing described in Section 3.2. 

**Figure 13:**
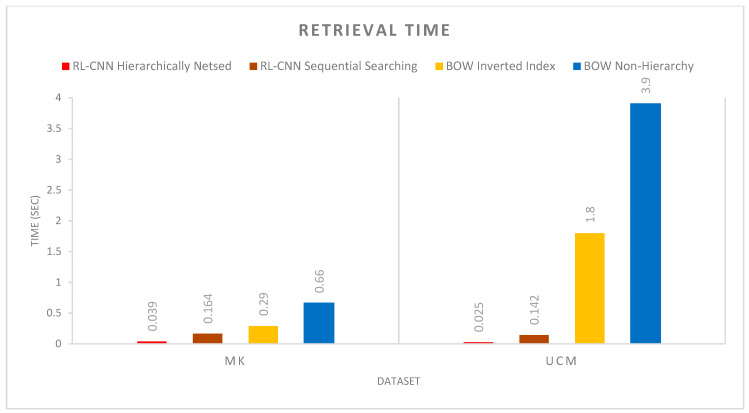
Execution times in seconds of the hierarchically nested indexing, inverted index, and non-hierarchical content-based image retrieval (CBIR), tested on Malaya–Kew and The University of California Merced datasets.

Figure 13 shows the execution time of CNN-hierarchically nested structure, CNN sequential searching, BOVW-inverted indexing technique, and BOVW-non-hierarchical retrieval method. The non-hierarchical method processes each image by scanning all images and computing similarity values between query images and all images. In contrast, the hierarchically nested indexing avoids comparing the query image with every image in the dataset by grouping similar images together and measures the similarity based on recursive density estimation. The inverted index technique also avoids performing a linear search over all images in the dataset, helping us to speed up the querying process. In the inverted index method, we query our inverted index to find images that contain the same visual word as the query image. Then, we only compare images in the dataset that contain a significant number of visual words as the query. 

As illustrated in Figure 13, the proposed hierarchical indexing method achieved the fastest retrieval performance. The average retrieval time of RL-CNN with hierarchical indexing scheme on the MK and UCM image datasets are 0.039 and 0.025 seconds, respectively. RL-CNN with sequential searching came second with 0.164 and 0.142 seconds, respectively. Nevertheless, this is an *O(N)* linear operation; thus, the execution time increases considerably in the sequential searching if the number of images increases to hundreds of thousands or millions. BOVW with inverted index and BOVW without indexing had the slowest retrieval time, with 0.29 and 1.8 seconds and 0.66 and 3.9 seconds in MK and UCM datasets, respectively. Moreover, the RL-CNN with sequential searching showed faster performance compared to the BOW with a similar structure. This can be justified since the RL-CNN computes a 2048-D feature vector, whereas BOW generates a 42,000-D feature representation (Section 4, the bag of visual words). As a result, it takes more time to compute similarity for every individual image in the dataset.

### 4.4. Discussion, Challenges and Future Work

Although the ability to retrieve digital images with relatively high accuracy and low computational efficiency was presented in this study, challenges remain in terms of optimizing the CNN model to derive better feature representations as well as developing a dynamic clustering technique to group similar images and form a hierarchically nested pyramid. 

In this study, we applied pre-existing network architecture pre-trained on data of some “related” domain and use it as feature extractor. However, if the testing dataset is not related to the training dataset that the pre-existing network is trained on (for example, hyperspectral or medical imagery), the pre-trained model will most likely have difficulty deriving discriminative features from the testing dataset. There is a type of transfer learning (TL) called fine-tuning that exists to leverage unlabeled data. Typically, these techniques attempt to pre-train the weights of the classification network, by iteratively training each layer to reconstruct the images. A combination of these techniques and pre-trained network is often used to improve convergence.

In terms of database structure and querying, the proposed indexing technique is an approximation based on visual similarity. Since the similarity measurement is based on data density estimation, the nearest neighbor will be either in the winning cluster or in the edge/border of another cluster, but in most cases in the same cluster, as the high accuracy achieved by the proposed methods presented in Section 4.2 indicates. However, the assumption can be modified from “winner takes all” to “few winners take all” to also include similar images fall into border clusters.

As shown, cluster/group feature vectors extracted from images based on their similarities will reduce the computational complexity of CBIR; however, in feature vectors with high dimension, data becomes very sparse and distance measures become increasingly meaningless, resulting in low performance of CBIR. Moreover, in some applications where the number of image categories is unknown, the difficulties become more vivid when the clustering method has a static nature and pre-defined structure, such as *K*-means. Future work will tackle dynamic clustering methods without the requirement of pre-defining the number of clusters in advance. The advantage of using such a model is that if new images are added to the dataset, the clustering images and forming the hierarchical structure will not be repeated from scratch. 

Another improvement will be adding relevant feedback which enables users to have more interaction with the system and provide feedback on the relevance of the retrieved images. The feedback can be used for learning and improving the performance of the CBIR.

## 5. Conclusions

The research scope for this paper focused on highly scalable and memory efficient image retrieval system. The aim was to overcome the limitations of conventional retrieval methods in the field of plant biology and remote sensing to significantly boost the retrieval performance in terms of accuracy and computational efficiency. The challenge was to preserve multi-dimensional and high discriminative image representations derived by the CNN model and still maintain the computational efficiency of the querying process. It is worth highlighting the following advantages of the proposed method:

- **Fast Retrieval time**: The proposed approach improves the retrieval process and is over 16 times faster than the traditional brute-force sequential searching which is vital for large-scale databases.
- **Scalability**: The model is constructed in a hierarchical structure. The feature indexing in a hierarchical form can handle a dynamic image database and can be easily integrated into the server-client architecture.
- **Unsupervised data mining**: The proposed technique does not require any prior knowledge of image repositories or any human intervention. However, in future work, human input/feedback can potentially improve the performance.
- **Recursive similarity measurement**: The similarity measurements are done recursively, which significantly reduces memory cost in high-scale multimedia CBIR systems.
- **Discriminative power for quantifying images**: Transfer learning is applied by utilizing a pre-trained deep neural network model merely as a feature extractor. The results indicate that the generic descriptors extracted from the CNNs are effective and powerful, and performed consistently better than conventional content-based retrieval systems.

Furthermore, although the visual content was the main focus of this study, integrating keywords and text to the CBIR pipeline can capture images’ semantic content and describe images which are identical by linguistic clues.

## Figures and Tables

**Table 1:** The retrieval accuracy *mAP* of convolutional neural network as a feature extractor (RL-CNN), bag of visual words (BOVW), and multiple fused global features (MFF) on Malaya–Kew (MK) and University of California Merced (UCM) datasets.

Dataset	Method	*mAP* (%)
MalayaKew	FE-CNN	88.1%
	BOVW	66.2%
	MFF	52.6%
UCM	FE-CNN	90.5%
	BOVW	86.2%
	MFF	69.8%

## Supplementary Materials

The following are available online at https://www.mdpi.com/2313-433X/5/3/33/s1, Additional file 1: The retrieval results of the CNN, BOW, and MFF methods tested on MK dataset. Additional file 2: The retrieval results of the CNN, BOW, and MFF methods tested on MK dataset. The results are presented in both HTML and PNG formats. It should be noted that the retrieval images in the HTML files will not be displayed due the missing link with the local hard drive stored the images; however, the class labels of the retrieved data can be validated in the HTML source code.

## Abbreviations

The following abbreviations are used in this manuscript:

CBIR	Content Based Image Retrieval
BOVW	Bag of Visual Words
SIFT	Scale Invariant Feature Transform
CNN	Convolutional Neural Network
DNN	Deep Neural Network
TL	Transfer Learning
RDE	Recursive Density Estimation
SPM	Spatial Pyramid Matching
LLC	Local Linear Constraint
FV	Fisher Vector
VLAD	Vector of Locally Aggregated Descriptors
MFF	Multiple Feature Fusion
IR	Information Retrieval
RL	Representation Learning
